# Self-Reported Prevalence of Functional Ankle Instability and Associated Factors Among Male Football Players in Southwestern Saudi Arabia

**DOI:** 10.3390/jcm15176634

**Published:** 2026-08-27

**Authors:** Abdulaziz Mohammed Y Alfaya, Ahmad Alanazi, Msaad Alzhrani, Sultan A. Alanazi, Ateef Mahamed

**Affiliations:** 1 Department of Physical Therapy, Aseer Rehabilitation Centre, Aseer General Hospital, Ministry of Health, Abha 62523, Saudi Arabia; amalfaya@moh.gov.sa; 2 Department of Physical Therapy & Health Rehabilitation, College of Applied Medical Sciences, Majmaah University, Al Majmaah 11952, Saudi Arabia; aalanazi@mu.edu.sa (A.A.); m.alzhrani@mu.edu.sa (M.A.); sa.alanazi@mu.edu.sa (S.A.A.)

**Keywords:** functional ankle instability, prevalence, associated factors, Ar-IdFAI, scales, field-position football players, southwestern Saudi Arabia

## Abstract

**Background/Objectives**: Rapid changes, jumping, and high-intensity movements are common among football players and are vulnerable to ankle sprains and functional ankle instability (FAI). This study aimed to determine the prevalence of FAI and assess the correlation with related factors among male football players in southwestern Saudi Arabia. **Methods**: A cross-sectional observational study was conducted among 364 male football players aged ≥18 years in southwestern Saudi Arabia. Participants completed the Arabic version of the Identification of Functional Ankle Instability (Ar-IdFAI) questionnaire. Descriptive statistics were used to estimate prevalence. Differences in Ar-IdFAI scores across playing positions were examined using analysis of covariance (ANCOVA), adjusted for age, body mass index (BMI), playing experience, and weekly training hours. Multivariable binary logistic regression was used to examine factors associated with IdFAI-defined probable FAI. **Results**: IdFAI-defined probable FAI was identified in 47.8% (174/364) of participants (95% CI: 42.7–52.9%). After adjustment for age, BMI, playing experience, and weekly training hours, playing position was not significantly associated with Ar-IdFAI score (ANCOVA, F(3,356) = 2.106, *p* = 0.099). In the exploratory participant-level logistic regression analysis, greater playing experience was associated with lower odds of Ar-IdFAI-defined probable FAI (OR = 0.93, 95% CI: 0.88–0.98, *p* = 0.018), whereas playing position, age, BMI, weekly training hours, and dominant limb were not statistically associated with the outcome. **Conclusions**: FAI is prevalent among all male field-position footballers, regardless of their position. Playing position was not independently associated with instability, whereas greater playing experience demonstrated an inverse association with functional ankle instability. Findings highlight the necessity for preventive and rehabilitation approaches targeting all players regardless of position.

## 1. Introduction

Football is a physically demanding game that involves various energy systems to support sporting performance [1,2,3]. There are several playing positions based on roles, orientation changes, propulsion, running with the ball, and other motor activities that take place during the game. These positions include defenders, midfielders, forwards, and goalkeepers [4,5]. Lateral ankle sprains are among the most common musculoskeletal injuries in physically active populations, and a substantial proportion of individuals who sustain ankle sprains subsequently develop chronic ankle instability characterised by recurrent sprains, instability, and functional limitations [6,7]. Ankle sprains frequently occur in sports and account for 16–40% of all trauma incidents associated with sports [8,9,10].

Approximately 40% of all ankle injuries and half of all ankle sprains are caused by sports, with basketball (41.1%), American football (9.3%), and soccer (7.9%) being the most common sports causing these injuries [7,11]. Approximately 85% of these ankle injuries are caused by lateral ankle sprains (LASs) [12]. In the United States, nearly 10% of the population experiences an ankle sprain at some point [6,13], and ankle sprains account for 15–45% of all football-related injuries nationwide [14,15]. In Tabuk, Saudi Arabia, the rate of ankle sprains is notably high, reaching 63.8% [16]. According to research, male football players have a high prevalence of ankle sprains, and 58.6% of all athletes experience re-injury. Published evidence indicates that approximately 85–90% of ankle sprains result from a sudden inversion mechanism, which places excessive strain on the lateral structures of the ankle [14,17].

The ‘Hertel model’ [18] was mainly employed to distinguish between mechanical and functional ankle instability (FAI), which is a subtype of chronic ankle instability (CAI) that results from repeated ankle sprains [18]. CAI encompasses recurrent ankle sprains, perceived instability, and episodes of giving way, and up to 40% of individuals who sustain ankle sprains may develop chronic instability symptoms [19]. Previous research has identified proprioceptive and neuromuscular impairments as factors associated with functional ankle instability [20]. CAI has also been associated with reduced ankle function, impaired quality of life, and long-term functional limitations in athletic populations, highlighting its clinical and functional significance [6]. Moreover, it has been observed that giving way occurred in 60% of reported incidents [21]. Long-term ankle instability is affected by various intrinsic and extrinsic factors, such as neuromuscular impairment and ligamentous injury, and biomechanical stress; these factors are strongly associated with the development of ankle instability [22]. Previous studies have reported an association between repeated ankle injuries and re-injuries and FAI among young football players [14,23]. Al-Mohrej reports that athletes with functional ankle instability (FAI) often experience limitations that prevent them from fully engaging in functional tasks or athletic activities [24]. Therefore, through assessment and intervention programs, we need to improve and focus on prevention, reduced risk factors and management [25]. Functional ankle instability can be assessed by physical examination, muscle strength tests, range of motion measurements, and radiographs. Most of the patient-reported outcome measures (PROMs) are currently used in sports [26,27] to evaluate FAI symptoms and function [23,28,29].

The IdFAI is widely recommended for clinical and research use and provides a validated measure of functional ankle instability severity and diagnosis based on instability symptoms and functional impairments [19,29,30]. The reliability of the IdFAI (English version) has been reported as 0.959 [21,29]. Additionally, the Ar-IdFAI has an acceptable reliability of 0.91 [5,31]. Researchers have differing opinions regarding the impact of different football positions on injury rates [32,33]. During a game, players are exposed to high-velocity contact with other team members, particularly during attacks. This involves continuous changes in direction and speed, which are associated with increased injury exposure [14,34]. Furthermore, recent evidence suggests that ankle instability represents a heterogeneous condition with variable clinical presentation, emphasizing the importance of population-specific investigation to improve diagnostic precision and targeted prevention strategies [21,35].

Although CAI is a broader clinical condition encompassing recurrent ankle sprains, episodes of giving way, perceived instability, and associated sensorimotor impairments, the present study operationally defined the outcome as self-reported FAI using the Arabic version of the Identification of Functional Ankle Instability (Ar-IdFAI) questionnaire. Throughout this manuscript, the term functional ankle instability refers specifically to instability classified according to the IdFAI.

Despite substantial football participation in Saudi Arabia, research evaluating the prevalence of FAI and associated factors among male football players according to playing position has not yet been conducted in the Kingdom of Saudi Arabia (KSA). Thus, football players must understand FAI, the associated factors that can potentially lead to ankle instability, and injuries associated with different football playing positions during play. The present study aimed to estimate the prevalence of IdFAI-defined probable functional ankle instability, examine differences in IdFAI scores across playing positions, and identify demographic, anthropometric, and playing-related factors associated with IdFAI-defined probable functional ankle instability among male football players in southwestern Saudi Arabia.

## 2. Materials and Methods

### 2.1. Study Design and Participants

A cross-sectional observational study was carried out by administering an Arabic version of the Identification of Functional Ankle Instability (Ar-IdFAI) questionnaire in southwestern Saudi Arabia from 25 January to 15 March 2025 to Saudi Arabian male football players across different playing positions. Player data were collected from the Saudi Arabian Football Federation, Riyadh, KSA (SAFF) and twenty-two sports clubs in southwestern Saudi Arabia: Abha Club (Abha, KSA), Damac Club (Khamis Mushait, KSA), Al-Areen Club (Dhahran Al-Janub, KSA), Al-Zaitoon Club (Sabt Al Alayah, KSA), Mahayel Club (Muhayil Asir, KSA), Jerash Club (Ahad Rufaidah, KSA), and Al-Musayf Club (Al-Musayf, KSA), Al-Fursan Club (Sarat Ubaidah, KSA), Al-Sarawat Club (Al-Namas, KSA), Bisha Club (Bisha, KSA), Al-Farouq Club (Al-Majardah, KSA), Almaa Club (Rijal Alma, KSA), Al-Bashaer Club (Al-Bashaer, KSA), Jazan Club (Jazan, KSA), Al-Tuhami Club (Jazan, KSA), Hottain Club (Samtah, KSA), Al-Yarmouk Club (Abu Arish, KSA), Al-Amjad Club (Sabya, KSA), Baish Club (Baish, KSA), Al-Sawari Club (Farasan, KSA), Al-Watan Club (Damad, KSA), and Fayfa Club, (Fayfa, KSA) [36]. The managers of all clubs were contacted, and the protocol was explained to facilitate data collection. Authorization to use the questionnaire in Saudi Arabia was obtained from the author of the Arabic translation of the instrument, Giza, Egypt [31] before applying for Institutional Review Board (IRB) approval. Ethical approval was obtained from the Majmaah University Research Ethics Committee, AlMajmaah, KSA (MUREC; approval no. MUREC-Jan.22/COM-2025/90) on 22 January 2025, and all participant recruitment, written informed consent, and data collection commenced after ethics approval.

A sample of 364 male football players across different playing positions (defenders, midfielders, forwards, and goalkeepers), aged ≥18 years, who had a minimum of 6 h of training exposure or more per week, gave their written informed consent and were playing without any clinical restrictions, were considered for participation in the study. Only male football players were included in the present study; therefore, the findings should not be generalized to female football players. Participants were excluded from the study to ensure that participants met the standards for reliable data collection and that confounding variables did not alter the outcomes. Participants were excluded if they had a systemic disorder that could impair balance, reflexes, muscle strength, or neuromotor control (e.g., multiple sclerosis or vestibular disorders), a lower-limb fracture within the previous two years, or a history of knee or hip injury involving the same leg. Data on ongoing medical treatment for ankle issues were obtained from the health registry of each club. The exclusion criteria were established in accordance with the guidelines of the International Ankle Consortium, Kentucky, USA [8]. Participants who were unwilling to provide written informed consent or who were unable to complete the questionnaires were also excluded.

### 2.2. Sample Size and Sampling Technique

Using the Raosoft sample size calculator, a target sample size of 364 participants was calculated based on an estimated population of approximately 6600 male football players in southwestern Saudi Arabia, with a 5% margin of error, 95% confidence level, and 50% response distribution. The estimated population was derived from approximately 300 eligible/registered male players per participating club across the 22 participating clubs (22 × 300 ≈ 6600). The available Saudi Arabian Football Federation (SAFF) information did not provide a region-specific count of eligible male players for southwestern Saudi Arabia; therefore, the 6600 figure was used as an operational estimate for sample-size planning rather than as an independently verified regional population denominator. Accordingly, the study sample can be considered a multicentre, position-stratified recruited sample, and the findings should be interpreted cautiously with respect to population representativeness. Participants were recruited across 22 football clubs; however, a club identifier was not retained in the final analytical dataset. Consequently, the extent of within-club correlation and club-specific sampling fractions could not be quantified retrospectively, and cluster-adjusted standard errors or multilevel analyses could not be performed. Accordingly, the inferential analyses were conducted at the participant level and are considered exploratory, with potential within-club correlation acknowledged as a source of uncertainty.

### 2.3. Description of Ar-IdFAI Questionnaire

To correctly identify individuals having or not having FAI, Simon and colleagues in 2012 developed a patient-reported outcome measure, the ‘Identification of Functional Ankle Instability (IdFAI) questionnaire,’ based on the relevant items from the two existing tools to evaluate FAI, the Ankle Instability Instrument (AII) and the Cumberland Ankle Instability Tool (CAIT), and integrated into the IdFAI to create a clear and concise tool for diagnosing FAI in individuals, Bloomington, USA [29]. The Arabic version of FAI was consequently cross-culturally translated and was noted to be an easy-to-apply, valid, and reliable scale. The IdFAI has demonstrated a specificity of 0.99, with scores ≤ 10 indicating that functional ankle instability is unlikely and scores ≥ 11 indicating that functional ankle instability is likely [31]. The elements of the IdFAI are classified into three components, making it an easy patient-reported outcome measure [31]. Component 1: Ankle instability history. The subjective sense of ankle instability is specifically addressed through items Q5, Q6, Q7, and Q10. It contains questions on how players feel unstable while playing sports or engaging in functional activities, how frequently their ankle gives way, when participants last experienced giving-way symptoms, the strategies they use to manage a twisted ankle. Component 2: First ankle sprain. The seriousness of an earlier ankle injury can be more accurately determined by answering these questions (Q1, Q2, Q3, and Q4). They ask about the number of ankle sprains the person has had, when their last ankle sprain occurred, when they needed weight-bearing assistance, and the ankle sprain grade determined by a medical professional. Component 3: Instability when engaging in normal tasks. This component contains inquiries (Q8 and Q9) regarding how long it takes for an ankle injury to heal and instability during daily living activities. The questionnaire consisted of ten questions. The scale’s first question evaluates the patient’s entire history of ankle sprains; it does not include the final score. The items in the last nine questions have a maximum score of five points and a minimum score of zero. The overall score was calculated by adding the scores of each of the nine questions. The final score may be as low as 0 points or as high as 37 points. According to the scale, a participant who has an overall score of 11 or more is considered to have ankle instability, whereas scores ≤ 10 indicate that functional ankle instability is unlikely.

### 2.4. Data Collection Procedure

Before data collection, the managers of the participating football clubs were contacted face-to-face, and the study protocol, including the anticipated time required to complete the study forms, was explained. The study was conducted across 22 football clubs in southwestern Saudi Arabia. The inclusion and exclusion criteria were explained to all potential participants, and eligibility was verified using the available health records maintained by the respective clubs. Eligible male football players representing the four playing positions—defenders, midfielders, forwards, and goalkeepers—were invited to participate. Participants who agreed to participate provided written informed consent before completing the study procedures. Demographic and anthropometric information was collected using a structured checklist, including age, height, weight, and body mass index (BMI). Football-related characteristics, including playing position, playing experience, weekly training hours, and dominant limb, were also recorded. Previous ankle injury, recurrent ankle injury, and episodes of the ankle giving way were recorded using predefined yes/no responses.

Participants completed the Ar-IdFAI questionnaire once. Participants were instructed to fill out the questionnaire according to the pre-specified index-ankle rule. The instructions given were “If you have symptoms in only one ankle, complete the questionnaire for that ankle. If you have symptoms in both ankles, complete two separate questionnaires—one for each ankle.” Participants with no ankle symptoms were instructed to complete the form for their dominant ankle. Participants selected the indexed ankle before reading the Ar-IdFAI items; then, the participants were asked to report the laterality of their perceived ankle symptoms, such as right ankle, left ankle, or both ankles. This laterality variable reflected the participant’s self-reported symptom distribution and did not represent independent IdFAI classifications of the two ankles. Laterality data were collected for descriptive purposes only and were not used to define bilateral FAI. The selection process employed in this study may result in a systematic bias for participants to receive higher (i.e., more unstable) IdFAI scores than if both ankles were scored and averaged or if a random ankle-selection rule had been used. Thus, the study cannot be compared.

The Ar-IdFAI total score was calculated by summing the nine scored items according to the instrument’s scoring algorithm. Participants were classified as IdFAI-defined probable FAI when the total score was ≥11, whereas scores ≤ 10 indicated that FAI was unlikely. All completed questionnaires were reviewed for completeness immediately after data collection. No participant included in the final analysis had missing responses to the Ar-IdFAI items required to calculate the total score. The collected data were subsequently compiled for statistical analysis.

### 2.5. Statistical Tools and Data Analysis

The data of this study were examined using the Statistical Package for the Social Sciences (SPSS), version 27.0 (IBM Corp., Armonk, NY, USA). The normality of continuous variables, such as age, BMI, playing experience (years), training hours per week, and Ar-IdFAI scores, was evaluated by using the Kolmogorov–Smirnov test. Variations in Ar-IdFAI scores across playing positions were examined using analysis of covariance (ANCOVA), adjusting for age, BMI, playing experience, and weekly training hours. ANCOVA was retained because the primary inferential objective was to estimate covariate-adjusted mean differences in Ar-IdFAI scores across the four playing-position groups. Although the observed Ar-IdFAI scores were non-normally distributed, the assessment of model adequacy focused on the ANCOVA assumptions, including linearity between continuous covariates and the outcome, homogeneity of regression slopes, homoscedasticity, and the distribution of standardized residuals. These assumptions were assessed using interaction terms, residual-versus-fitted plots, and Q–Q plots before the final model was interpreted. Categorical variables were expressed as frequencies and percentages.

Prevalence of IdFAI-defined functional ankle instability (FAI) was examined by using frequency and percentage analysis based on the validated IdFAI classification cutoff score (≥11). Variations in Ar-IdFAI scores across playing positions were examined by using analysis of covariance (ANCOVA), adjusting for potential confounding variables including age, BMI, playing experience, and weekly training hours. Before ANCOVA, model assumptions were assessed, including linearity between continuous covariates and the dependent variable, homogeneity of regression slopes by testing interaction terms, homoscedasticity through visual inspection of residual-versus-fitted plots, and approximate normality of standardized residuals using Q–Q plots. The assumptions were considered acceptable before conducting the final model. Before the regression analysis, multicollinearity diagnostics were conducted to examine independent variables. Variance inflation factor (VIF) and tolerance values were evaluated, and all variables met acceptable thresholds (VIF < 5, tolerance > 0.20), verifying the absence of multicollinearity.

Binary logistic regression was performed using IdFAI-defined probable functional ankle instability status (0 = absent, 1 = present) as the dependent variable. Goalkeepers were used as the reference category for playing position, and right-limb dominance served as the reference category for dominant limb. All 364 participants had complete data for the variables included in the model; therefore, listwise deletion did not reduce the analysed sample (N = 364). The linearity of the logit for age, BMI, playing experience, and weekly training hours was assessed using the Box–Tidwell approach. No statistically significant evidence of departure from linearity was identified for any of the continuous predictors. The correlation between age and playing experience was assessed using Pearson’s correlation coefficient. Multicollinearity was evaluated using variance inflation factors (VIFs) and tolerance statistics. VIFs were 2.26 for age, 1.27 for BMI, 2.08 for playing experience, and 1.03 for weekly training hours, with corresponding tolerance values of 0.443, 0.789, 0.480, and 0.975, respectively. Odds ratios (ORs), regression coefficients (β), standard errors (SEs), and 95% confidence intervals (CIs) were reported.

Because participants were recruited from 22 football clubs, observations may have been correlated within clubs. However, a club identifier was not retained in the final analytical dataset; therefore, cluster-adjusted standard errors, generalized estimating equations, multilevel models, intraclass correlation coefficients, and design-effect estimates could not be calculated retrospectively. Accordingly, the ANCOVA and binary logistic regression analyses were conducted at the participant level and are considered exploratory, with the possibility of residual within-club correlation acknowledged in the interpretation of confidence intervals and *p*-values.

Variables representing previous ankle injury, recurrent injury, and giving-way episodes were collected for descriptive purposes but were not included in the multivariable regression models because these variables overlap conceptually with the Ar-IdFAI outcome measure and could introduce criterion contamination or circular associations.

## 3. Results

### Participants

Overall, 364 male football players across different playing positions in different categories were recruited from southwestern Saudi Arabia. The demographic and injury-related characteristics of the participants are presented in Table 1, while position-specific FAI prevalence is presented in Table 2a. The demographic characteristics did not show a normal distribution. Hence, they were presented as medians with interquartile ranges and ranges (Table 1). Injury-related characteristics are summarized at the end of Table 1. Previous ankle injury, recurrent injury, and episodes of giving way were recorded for descriptive characterization of the study population. Dichotomous or nominal variables were expressed as frequencies and percentages. Among the 364 male football players included in this study, 47.8% (174/364) reported FAI, while 190 players (52.2%) did not meet the criteria for instability. Among the 364 participants, 174 (47.8%; 95% CI: 42.7–52.9%) were classified as IdFAI-positive for probable FAI, while 190 (52.2%) did not meet the classification threshold. Position-specific prevalence was highest among forwards (61.3%; 95% CI: 48.8–72.6%), followed by midfielders (48.2%; 95% CI: 40.1–56.4%), defenders (42.6%; 95% CI: 33.4–52.3%), and goalkeepers (41.7%; 95% CI: 29.9–54.4%). Of the 174 IdFAI-positive participants, 68 were midfielders, 43 were defenders, 38 were forwards, and 25 were goalkeepers (Table 2a).

Of the 174 participants classified as FAI-positive, 43 were defenders, 68 were midfielders, 38 were forwards, and 25 were goalkeepers (Table 2a).

Self-reported symptom laterality among the 174 participants with an Ar-IdFAI-positive index ankle is presented in Table 2b. Among these participants, 81 (46.6%) reported symptoms affecting the right ankle, 40 (23.0%) reported symptoms affecting the left ankle, and 53 (30.5%) reported symptoms affecting both ankles. These laterality responses were collected separately from the Ar-IdFAI score and were used for descriptive purposes only. The numbers and percentages of right-sided, left-sided, and bilateral involvement have been reported to provide a clearer description of the pattern of ankle instability within the study population.

Previous ankle injury was reported by 306 participants (84.1%), while 175 (48.1%) reported experiencing a recurrent ankle injury. Episodes of the ankle giving way were reported by 178 participants (48.9%) (Table 1). These variables were collected to describe the clinical characteristics of the study population but were not included in the multivariable regression analyses because they overlap conceptually with the IdFAI outcome measure and could introduce criterion contamination.

Mean Ar-IdFAI score for the total sample was 10.11 ± 7.57. Position-specific mean Ar-IdFAI scores were highest among forwards (11.77 ± 8.04), followed by midfielders (10.55 ± 7.59), defenders (9.35 ± 7.26), and goalkeepers (8.62 ± 7.25) (Table 2a). After adjustment for age, BMI, playing experience, and weekly training hours, the overall effect of playing position on Ar-IdFAI score was not statistically significant (F(3,356) = 2.106, *p* = 0.099, partial η^2^ = 0.017). An exploratory adjusted contrast suggested higher Ar-IdFAI scores among forwards than goalkeepers (B = 3.137, 95% CI: 0.90–3.88, *p* = 0.095); however, this isolated contrast was interpreted cautiously given the non-significant omnibus test. Multicollinearity diagnostics were conducted before regression analysis. All predictor variables demonstrate acceptable tolerance (>0.20) and variance inflation factor values (<5), indicating absence of multicollinearity and confirming suitability for multivariable modelling.

Binary logistic regression analysis was carried out to examine the relation between demographic and playing characteristics and functional ankle instability status (Table 3).

All 364 participants had complete data for the variables included in the logistic regression, and no observations were excluded through listwise deletion. Age was moderately to strongly correlated with playing experience (r = 0.697, *p* < 0.001); however, the observed VIF and tolerance values did not indicate problematic multicollinearity. The Box–Tidwell assessment showed no statistically significant evidence of nonlinearity in the logit for age, BMI, playing experience, or weekly training hours (all *p* > 0.05).

Although the regression model demonstrated acceptable calibration according to the Hosmer–Lemeshow test, the relatively low Nagelkerke R^2^ indicates that the measured variables accounted for only a limited proportion of the variability in functional ankle instability. This suggests that additional biomechanical, neuromuscular, injury-history, and training-load factors not measured in the present study may contribute to instability risk. The model showed no evidence of poor calibration according to the Hosmer–Lemeshow test (χ^2^ = 2.924, *p* = 0.939); however, the Nagelkerke R^2^ of 0.053 indicated that the measured predictors accounted for only a limited proportion of the variability in IdFAI-defined probable functional ankle instability. Playing position was not significantly associated with functional ankle instability (overall *p* = 0.293). Compared to goalkeepers, defenders (OR = 1.05, 95% CI: 0.53–2.08, *p* = 0.887), midfielders (OR = 1.23, 95% CI: 0.66–2.30, *p* = 0.516), and forwards (OR = 1.87, 95% CI: 0.90–3.88, *p* = 0.095) did not show statistically significant differences in instability odds. In the exploratory participant-level logistic regression analysis, greater playing experience was associated with lower odds of IdFAI-defined probable FAI (β = −0.067, SE = 0.028, adjusted OR = 0.94, 95% CI: 0.88–0.98, *p* = 0.018). Playing position, age, BMI, weekly training hours, and dominant limb were not statistically associated with IdFAI-defined probable FAI.

## 4. Discussion

This study extends the existing Saudi Arabian evidence on IdFAI-defined probable functional ankle instability by examining a multicentre sample of 364 male football players recruited from 22 clubs in southwestern Saudi Arabia, with specific evaluation of playing-position distributions and associations with demographic, anthropometric, and playing-related factors. Among the total recruited sample of 364 football players, 47.8% (95% CI: 42.7–52.9%) (174/364) reported perceived FAI based on the validated IdFAI classification criteria; this prevalence highlights that functional ankle instability is a common condition among football players in this region and aligns with previously reported prevalence rates in athletic populations. Although midfielders accounted for the largest number of players with functional ankle instability because they comprised the largest positional subgroup in the cohort, the highest position-specific prevalence was observed among forwards. This distinction underscores the importance of interpreting prevalence within each playing position rather than relying solely on the absolute number of affected players. A recent Saudi Arabian study using the same Ar-IdFAI questionnaire reported a 44.53% prevalence of FAI among 128 male volleyball players from the Qassim region [37]. Although the sport and study setting differed from the present football cohort, the comparable prevalence provides contextual evidence that self-reported ankle instability is common among Saudi Arabian athletes. Another recent Saudi Arabian study of 218 male football players in Madinah Province reported a similar overall FAI prevalence of 47.7% using the same Ar-IdFAI questionnaire [38]. However, that study examined differences across age groups and used two-stage cluster sampling and multivariate linear regression, whereas the present study focused on playing position among male football players recruited from 22 clubs in southwestern Saudi Arabia and used position-stratified recruitment, ANCOVA of continuous Ar-IdFAI scores, and multivariable logistic regression of IdFAI-defined probable FAI status. These differences extend the available Saudi Arabian evidence by examining playing-position patterns and associated factors in a different regional football population. The present study should be considered complementary to previous Saudi Arabian studies using the Ar-IdFAI rather than an extension of the same dataset: prior work examined male volleyball players in the Qassim region [37] and age-group differences among male football players in Madinah [38], whereas the present study examined playing-position patterns and associated factors in a separate football cohort from southwestern Saudi Arabia.

The present study found that there was no statistically significant association between playing position and functional ankle instability state after adjusting for age, BMI, athletes’ playing experience, and weekly training hours. Although forwards had the highest observed mean Ar-IdFAI score, the overall adjusted effect of playing position was not statistically significant. An exploratory adjusted contrast suggested higher Ar-IdFAI scores among forwards than goalkeepers; this isolated contrast should be interpreted cautiously and does not establish an overall positional difference in Ar-IdFAI scores. Despite differences in movement strategies, including rapid directional changes, sprinting [39,40], kicking, jumping, defensive movements, and physical contact, players across positions may experience different patterns of ankle loading. However, biomechanical loading was not measured in the present study, and its relationship with IdFAI-defined probable FAI could not be evaluated.

Prior studies have reported inconsistent prevalence rates of ankle instability across playing positions, with goalkeepers and defenders often showing higher rates than other positions. This suggests that position-specific differences in movement patterns, physical contact, and tactical demands may contribute to variation in injury patterns and susceptibility to ankle instability [5]. Conversely, the results of the present study did not find a significant association between age, BMI and FAI, as also supported by a recent study [37] or between playing position and ankle instability status after adjustment for relevant covariates, indicating that the demographic and training-related variables examined in this study were not statistically associated with IdFAI-defined probable FAI. The present study did not assess cumulative injury mechanisms or biomechanical factors and therefore cannot determine the mechanisms underlying the observed prevalence or associations. This variation possibly relates to differences in study populations, sample sizes, or methodological approaches used across the studies, along with disparity in assessment tools and statistical analysis methods. The overall finding of this study was consistent with (47.8%) prior reported rates in football players. Similarly, a recent study using the Turkish version of the IdFAI questionnaire reported a comparable prevalence of 46.3%, lending support to the external validity of our findings. However, other research studies also found a similar prevalence of 66.66% using the Cumberland Ankle Instability Tool (CAIT) [9,41,42], 41% in football players [14] and 57.74% among basketball players [43,44]. These variations in prevalence estimates across the studies may be attributed to differences in study methodology assessments tools used to assess ankle instability among the players.

The Ar-IdFAI questionnaire used in this research study is a validated and reliable instrument for assessing functional ankle instability and has been suggested for use in both clinical and research settings [5,29,31]. Conversely, assessment instruments such as the CAIT, the Modified Ankle Instability Questionnaire, and the IdFAI provide a more comprehensive evaluation of instability symptoms and functional limitations, thereby enhancing the reliability of prevalence estimates within athletic populations.

Earlier studies also show similarity to this study in that FAI was not associated with age, BMI, weekly training hours and limb dominance. Weekly training hours and limb dominance were also not significantly associated with ankle instability [14,45]. In the present analysis, age, BMI, weekly training hours, and limb dominance were not statistically associated with IdFAI-defined probable FAI. These findings should not be interpreted as evidence that these factors have no relationship with ankle instability. Previous research has implicated neuromuscular and biomechanical factors in ankle instability; however, these factors were not measured in the present study and therefore cannot be evaluated as explanations for our findings. Neuromuscular adaptation, proprioceptive function, and movement strategies may plausibly contribute to differences in ankle function among athletes, but these mechanisms were not assessed in the present study. In the exploratory participant-level analysis, greater playing experience was associated with lower odds of IdFAI-defined probable FAI. This association remained evident after adjustment for the demographic, anthropometric, playing-related, and dominant-limb variables included in the model. However, because of the cross-sectional design, the direction of this association cannot be established. One possible explanation is a healthy-athlete survivor effect, whereby players with persistent ankle instability may be more likely to reduce participation or discontinue competitive football, leaving a cohort of more experienced players with a lower observed prevalence of instability. Prospective longitudinal studies are needed to determine whether playing experience truly influences the development of functional ankle instability.

Although previous injury, re-injury, and giving-way episodes were recorded, these variables were not entered into the regression model because they form part of the clinical construct underlying functional ankle instability as measured by the Ar-IdFAI.

The multivariable logistic regression model showed no evidence of poor calibration according to the Hosmer–Lemeshow test; however, the low Nagelkerke R^2^ indicated that the variables included in the model explained only a limited proportion of the variability in IdFAI-defined probable functional ankle instability. This indicates that additional factors not measured in the present study may account for variation in IdFAI-defined probable FAI. Future studies should incorporate these variables to improve risk prediction and develop comprehensive prevention strategies.

Earlier biomechanical and clinical research has found that ankle instability is associated with neuromuscular limitation, proprioceptive deficits, ligamentous laxity, and altered muscle activation patterns, notably involving the peroneal longus and brevis muscles and a weak lateral ankle ligament complex [46,47,48,49]. Frequent ankle loading, insufficient rehabilitation, and cumulative microtrauma contribute further to the development of FAI among the athletes. Consistent with earlier literature, the present findings are that functional ankle instability is common among football players and is not associated with demographics and specific playing positions. Positional roles require varying movement demands and biomechanical loading. Earlier systematic reviews have also demonstrated mixed findings regarding the association between playing position and injury risk, suggesting that various interacting factors can lead to ankle instability [5,12,14,32,33].

Understanding the prevalence and associated factors of IdFAI-defined probable FAI may help inform future research and clinical assessment strategies in football populations and may potentially enhance athlete performance and long-term joint health, thereby reinforcing the value of evidence-based prevention and rehabilitation strategies across musculoskeletal conditions. The present study, however, did not evaluate the effectiveness of screening, rehabilitation, or injury-prevention interventions. Further research is needed to combine biomechanical, neuromuscular, and clinical assessments to identify modifiable variables and optimize prevention strategies in football populations.

From a clinical perspective, validated patient-reported outcome measures may be useful for identifying players who warrant further assessment; however, the present study did not evaluate the effectiveness of screening, neuromuscular training, proprioceptive training, balance training, or rehabilitation interventions. These findings contribute to understanding the epidemiological profile of functional ankle instability among football players. Future prospective studies are required to determine the temporal and causal relationships underlying these associations and to evaluate whether targeted interventions improve clinical and functional outcomes.

### Limitations of the Study

This study has a few methodological and interpretational limitations. First, its cross-sectional design prevents causal inference, and the observed association between playing experience and lower odds of functional ankle instability may be influenced by reverse causation or a healthy-athlete survivor effect. Second, FAI was assessed using the self-reported Ar-IdFAI questionnaire without objective clinical, biomechanical, or functional assessment, which may introduce recall and reporting bias and limit classification precision. The participant-level prevalence estimate is based on a ‘‘study-specific index-ankle rule’’ rather than the standard bilateral IdFAI administration and should not be directly compared to prevalence estimates derived from studies using per-ankle scoring.

The questionnaire was completed with reference to each participant’s most symptomatic ankle; therefore, ankle-specific right–left comparisons and bilateral analyses were not possible, although laterality was documented descriptively. In addition, club-level sampling-frame counts, selection fractions, and the exact randomization procedures were not retained, preventing retrospective verification of equal selection probabilities and detailed assessment of non-response. Therefore, the sample should be interpreted as a multicentre, position-stratified recruited sample rather than as a fully verifiable probability sample. Third, the study was powered primarily to estimate overall prevalence and not for positional subgroup comparisons or multivariable regression; therefore, findings involving smaller positional groups should be interpreted cautiously. The response rate could not be calculated because the number of eligible players approached at each club was not prospectively recorded, and a participant flow diagram could not be constructed retrospectively. In addition, competitive level or league division was not collected; therefore, the findings cannot be interpreted according to specific levels of football competition, and generalisability across competitive levels remains uncertain.

Fourth, the regression model explained only a modest proportion of the variability in FAI, and several potentially relevant factors were not assessed, including rehabilitation history, competitive level or league division, footwear, playing surface, exposure to injury-prevention programs, and other biomechanical, neuromuscular, and psychological factors. Previous ankle injury, recurrent injury, and giving-way episodes were collected descriptively but were not included in the regression model because of their conceptual overlap with the Ar-IdFAI outcome and the potential for criterion contamination. Finally, participants were exclusively male football players from southwestern Saudi Arabia; therefore, the findings may not be generalizable to female athletes, other regions, age groups, recreational players, or different competitive settings. In addition, participants were recruited from multiple clubs, but clustering by club was not explicitly modelled, so residual within-club correlation cannot be excluded.

## 5. Conclusions

IdFAI-defined probable functional ankle instability was common among male football players across all playing positions, with an overall prevalence of 47.8%. Playing position was not independently associated with IdFAI-defined probable functional ankle instability after adjustment for the measured covariates. Greater playing experience was associated with lower odds of IdFAI-defined probable functional ankle instability; however, the cross-sectional design prevents causal interpretation of this association. The findings support further prospective research examining clinical, biomechanical, neuromuscular, and injury-related factors that may contribute to ankle instability and evaluating the effectiveness of targeted prevention and rehabilitation strategies.

## Figures and Tables

**Table 1:** Participant characteristics and functional ankle instability status (N = 364).

Variable		Value	
Continuous Variables		Median (IQR), Range	
Age (years)		32.00 (11.00), 18–38	
Body mass index (kg/m^2^)		23.48 (4.16), 16.39–34.37	
Playing experience (years)		12.00 (10.00), 2–21	
Weekly training hours		6.00 (1.00), 6–14	
Ar-IdFAI score		10.00 (13.00), 0–28	
**Categorical variables, n (%)**			
**Playing position:**			
Defenders		101 (27.7)	
Midfielders		141 (38.7)	
Forwards		62 (17.0)	
Goalkeepers		60 (16.5)	
**Functional ankle instability**			
Present		174 (47.8%)	
Absent		190 (52.2%)	
**Injury Characteristics**			
**Variable**	**Category**	**n**	**%**
**Previous ankle injury**	Yes	306	84.1
	No	58	15.9
**Re-injury**	Yes	175	48.1
	No	189	51.9
**Giving-way episodes**	Yes	178	48.9
	No	186	51.1

Note. Values are presented as median (interquartile range) and range for continuous variables and frequency (percentage) for categorical variables. Ar-IdFAI = Arabic Identification of Functional Ankle Instability.

**Table 2:** (**a**) Descriptive Ar-IdFAI scores and position-specific prevalence of IdFAI-defined probable FAI (N = 364). (**b**) Self-reported symptom laterality among participants with an Ar-IdFAI-positive index ankle (n = 174).

(**a**)						
**Playing Position**	**n**	**Mean**	**SD**	**FAI Positive (n)**	**Prevalence (%)**	**95% CI**
Defender	101	9.35	7.26	43	42.6	33.4–52.3
Midfielder	141	10.55	7.59	68	48.2	40.1–56.4
Forward	62	11.77	8.04	38	61.3	48.8–72.6
Goalkeeper	60	8.62	7.25	25	41.7	29.9–54.4
Total	364	10.11	7.57	174	47.8	42.7–52.9
(**b**)						
**Laterality**		**n**		**n (%) Among IdFAI-Positive Participants (n = 174)**		
Right		81		46.6		
Left		40		23.0		
Bilateral		53		30.5		
Total		174		100.0		

Note: Ar-IdFAI = Arabic Identification of Functional Ankle Instability. Values are presented as mean and standard deviation. Prevalence was calculated as the number of participants with IdFAI-defined probable FAI divided by the total number of participants within each playing position. Ninety-five per cent confidence intervals were calculated using the Wilson score method. Values shown as mean ± SD are unadjusted descriptive statistics.

**Table 3:** Binary logistic regression analysis of factors associated with FAI (N = 364).

Variable	β	SE	Adjusted OR	95% CI	*p*
Defender vs. Goalkeeper	0.095	0.346	1.10	0.53–2.08	0.887
Midfielder vs. Goalkeeper	0.328	0.317	1.39	0.66–2.30	0.516
Forward vs. Goalkeeper	0.717	0.376	2.05	0.90–3.88	0.095
Age (years)	0.028	0.025	1.03	0.99–1.09	0.122
BMI (kg/m^2^)	−0.013	0.037	0.99	0.93–1.08	0.916
Playing experience (years)	−0.067	0.028	0.94	0.88–0.98	0.018 *
Weekly training hours	0.150	0.137	1.16	0.93–1.39	0.218
Left vs. Right dominant limb	−0287	0.257	0.75	0.68–1.86	0.656

Note: OR = odds ratio; CI = confidence interval; β = regression coefficient; SE = standard error. Goalkeepers were the reference category for playing position, and right-limb dominance was the reference category for dominant limb. ORs were mutually adjusted for playing position, age, BMI, playing experience, weekly training hours, and dominant limb. * = significance.

## Data Availability

The dataset that was analysed or the results presented in this study are available on reasonable request from the corresponding author.

## Abbreviations

The following abbreviations are used in this manuscript:

FAI	Functional ankle instability
BMI	Body mass index
CAI	Chronic ankle instability
US	United States
IdFAI	Identification of Functional Ankle Instability
Ar-IdFAI	Arabic version of the Identification of Functional Ankle Instability
ANCOVA	Analysis of covariance
PROM	Patient-reported outcome measure
KSA	Kingdom of Saudi Arabia
AII	Ankle Instability Instrument
CAIT	Cumberland Ankle Instability Tool
IBM	International Business Machines
NY	New York
USA	United States of America
IRB	Institutional Review Board
IQR	Interquartile range
SAFF	Saudi Arabian Football Federation
MUREC	Majmaah University Research Ethics Committee
SPSS	Statistical Package for the Social Sciences
VIF	Variance inflation factor
SE	Standard error
OR	Odds ratio
CI	Confidence interval
